# Genome wide search to identify reference genes candidates for gene expression analysis in *Gossypium hirsutum*

**DOI:** 10.1186/s12870-019-1988-3

**Published:** 2019-09-14

**Authors:** P. K. Smitha, K. Vishnupriyan, Ananya S. Kar, M. Anil Kumar, Christopher Bathula, K. N. Chandrashekara, Sujan K. Dhar, Manjula Das

**Affiliations:** 1 Department of Biotechnology, Research and Development Centre, Bharathiar University, Coimbatore, Tamil Nadu 641 046 India; 2 Beyond Antibody LLP, S-005 Krishna Greens, Krishna Temple Road, Dodda Bomasandra, Bangalore, Karnataka 560 097 India; 3 Tumor Immunology Program, DSRG1, MSCTR, MSMF, Mazumdar Shaw Medical Centre, 8th floor, Narayana Health City, Bommasandra, Bangalore, Karnataka 560 099 India; 4 School of Bio Sciences & Technology, Vellore Institute of Technology, Vellore, Tamil Nadu 632 014 India; 5 Division of Plant Physiology and Biotechnology, UPASI Tea Research Foundation, Tea Research Institute, Nirar Dam, Valparai, Coimbatore, Tamil Nadu 642 127 India

**Keywords:** Reference gene, qPCR, *Gossypium hirsutum*, Transgenic, Data science, Cotton

## Abstract

**Background:**

Cotton is one of the most important commercial crops as the source of natural fiber, oil and fodder. To protect it from harmful pest populations number of newer transgenic lines have been developed. For quick expression checks in successful agriculture qPCR (quantitative polymerase chain reaction) have become extremely popular. The selection of appropriate reference genes plays a critical role in the outcome of such experiments as the method quantifies expression of the target gene in comparison with the reference. Traditionally most commonly used reference genes are the “house-keeping genes”, involved in basic cellular processes. However, expression levels of such genes often vary in response to experimental conditions, forcing the researchers to validate the reference genes for every experimental platform. This study presents a data science driven unbiased genome-wide search for the selection of reference genes by assessing variation of > 50,000 genes in a publicly available RNA-seq dataset of cotton species *Gossypium hirsutum*.

**Result:**

Five genes (*TMN5, TBL6, UTR5B, AT1g65240* and *CYP76B6*) identified by data-science driven analysis, along with two commonly used reference genes found in literature (*PP2A1 and UBQ14*) were taken through qPCR in a set of 33 experimental samples consisting of different tissues (leaves, square, stem and root), different stages of leaf (young and mature) and square development (small, medium and large) in both transgenic and non-transgenic plants. Expression stability of the genes was evaluated using four algorithms - geNorm, BestKeeper, NormFinder and RefFinder.

**Conclusion:**

Based on the results we recommend the usage of *TMN5* and *TBL6* as the optimal candidate reference genes in qPCR experiments with normal and transgenic cotton plant tissues. *AT1g65240* and *PP2A1* can also be used if expression study includes squares. This study, for the first time successfully displays a data science driven genome-wide search method followed by experimental validation as a method of choice for selection of stable reference genes over the selection based on function alone.

**Electronic supplementary material:**

The online version of this article (10.1186/s12870-019-1988-3) contains supplementary material, which is available to authorized users.

## Background

Real time quantitative PCR (qPCR) is the most reliable, accurate and cost-effective technique for studying differential gene expression [1]. This technique has an edge over other commonly employed methods in gene expression like Northern blot hybridization and RT-PCR (reverse transcription polymerase chain reaction), owing to its high sensitivity, specificity, accuracy and broad dynamic range [2, 3]. However, the major pitfalls affecting qPCR data are the variability in multiple factors such as quality and integrity of RNA samples, the efficiency of cDNA synthesis and PCR efficiency across experiments [4]. An appropriate normalization strategy like the selection of good reference genes is crucial to arrive at biologically meaningful gene expression results from qPCR experiments [5].

A good reference gene should have a constant level of expression across various experimental parameters [6, 7] or between cells of different tissues [8]. Traditionally used reference genes for qPCR studies were carryovers from semi-quantitative methods [9]. Often the expression level of many of these genes was found to be very high leading to faulty normalization for low expressing target genes in highly sensitive qPCR experiments [10]. It was cautioned to discontinue their usage for qPCR, as numerous studies began to demonstrate that the transcript levels of these genes can vary considerably under different physiological conditions [11].

The availability of genome-wide expression data through high throughput experiments like microarray has helped to identify stable genes that could be used for normalization in qPCR experiments [1]. Among the initial work done [12], the main focus was on evaluating a set of known reference gene candidates for the stability of expression using several normalization algorithms - geNorm [13], NormFinder [14] and BestKeeper [15]. However, some researchers also tried assessing gene stability using the bioinformatics approach [16], statistical measures like the coefficient of variation (CV) [17] and difference in DNA entropies in promoters driving the expression of reference and tissue specific genes [18]. Assuming that true reference genes should follow Normal distribution across samples, it was attempted to discover reference genes for expression studies in Soybean using CV and *p*-value from a normality test [19]. In another attempt, an automated workflow called findRG [20] was proposed to find reference genes in different plant species and human cancers using the CV as the primary measure. However, the approach of using high throughput transcriptome sequencing (RNA-seq) data to identify genes with least variation has become quite popular off late as evident from a number of studies in diverse organisms as human [21], apple [22], molusc [23] and *C. savignyi*, a marine organism [10].

Cotton is one of the most economically important cultivated crops. It is the major source of natural fiber for the textile industry and thus an important target for genetic modification. Transgenic technology has been applied to cotton for improving the agronomic traits, tolerance to insects, resistance to herbicides and fiber qualities [24, 25]. In this study, we have used Bollgard II (Monsanto) transgenic cotton that contains two *Bacillus thuringiensis* genes *Cry1Ac* and *Cry2Ab* proven to have good insecticidal efficacy against Lepidopteran larvae (cotton bollworm: *Helicoverpa armigera*). Numerous studies have focused on identifying the most suitable reference genes for different cotton species under biotic and abiotic stresses [11, 24, 26–36]. However, the study on the effect of expression of a transgene becomes very important, as various studies have indicated an altered expression of endogenous genes because of the expression of transgenes in both plants [37, 38] and animals [39]. Thus, the influence of the transgene on the expression levels of the endogenous genes, especially those used as reference genes needs to be thoroughly evaluated [39] before the start of a study.

In this study, we have employed an unbiased genome-wide search from a publicly available gene expression dataset for cotton to identify potential reference gene candidates to finally select the most stable genes for gene expression analysis in cotton. We have addressed expression stability of the potential candidate reference genes to be used in both non-transgenic and transgenic lines of *G. hirsutum* under various experimental conditions comprising of different tissues (leaves, stem and squares), age categories (1 to 3 month old plant), developmental stages of leaves (young and mature leaves) and square (small, medium and large squares). A data-driven analysis approach complemented with experimental validation used in this study can be extended to other scientific model systems with a large number of data.

## Results

### Selection of candidate genes

**Fig. 1:**
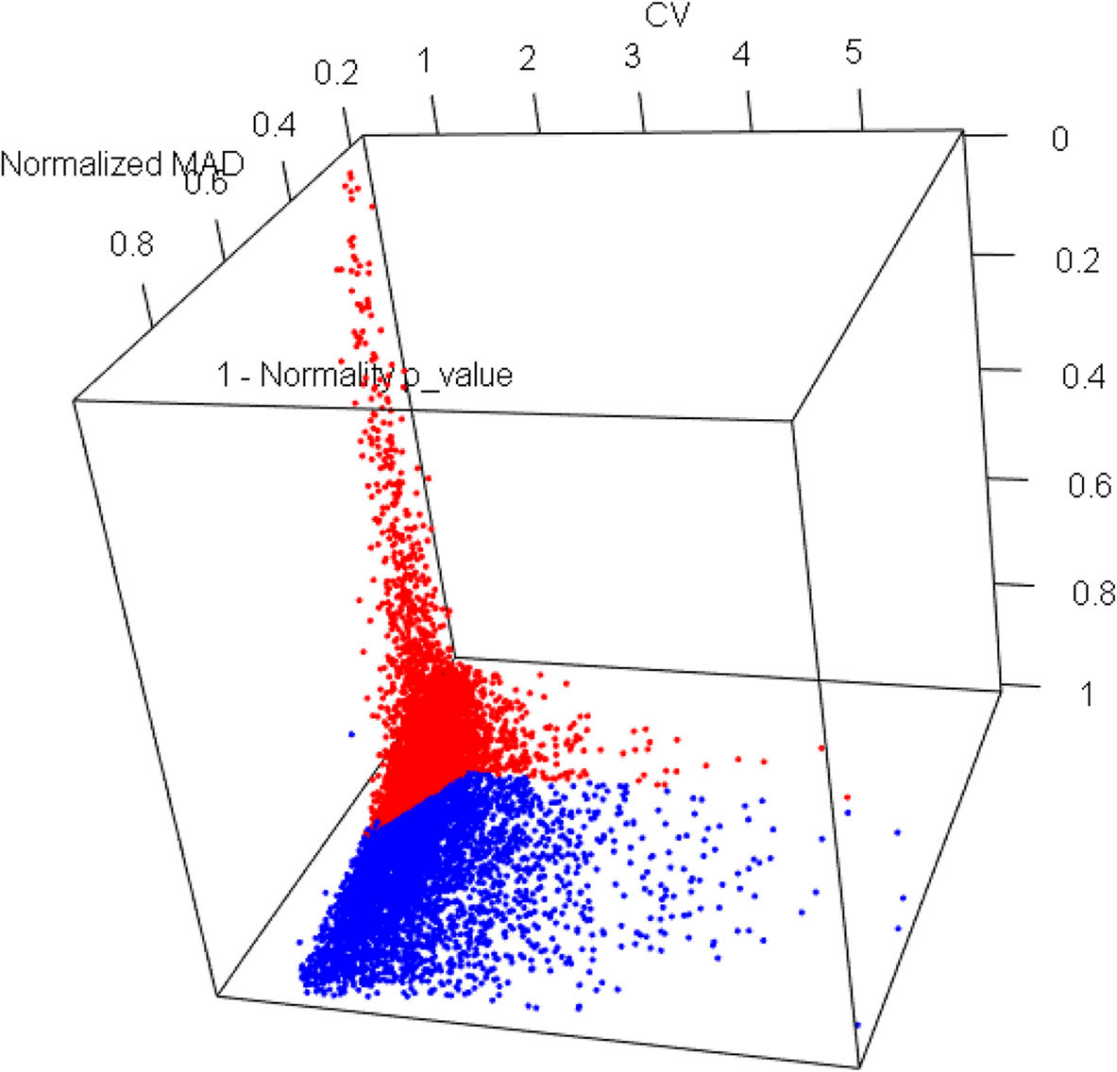
Cluster of genes in the three-dimensional space of CV, MAD and 1-p obtained using the PAM method. Genes marked in red represent cluster #1

Candidate reference genes were chosen in an unbiased manner from the publicly available cotton FGD dataset (www.cottonfgd.org) containing RNA-seq FPKM values for 66,577 genes. Out of this set only 51,272 genes could be mapped to a gene name from JGI annotation available as a part of the same dataset. From this annotated set, 11,137 genes were eliminated as low-expressing genes (median FPKM ≤0) and the analysis was carried out using the remaining 40,135 genes. Silhouette analysis indicated that only two clusters were most optimal for the analysis (Additional file 3). A representation of the two clusters in (CV, MAD, 1-p) hyperspace is shown in Fig. 1 with the details given in Table 1.

**Table 1:** Medoid Z scores of the clusters

Cluster	Number of genes	Z-score values for the medoid in each cluster		
		CV	MAD	1-p
1	5973	−0.599	−0.676	0.221
2	4061	0.426	0.933	0.223

**Fig. 2:**
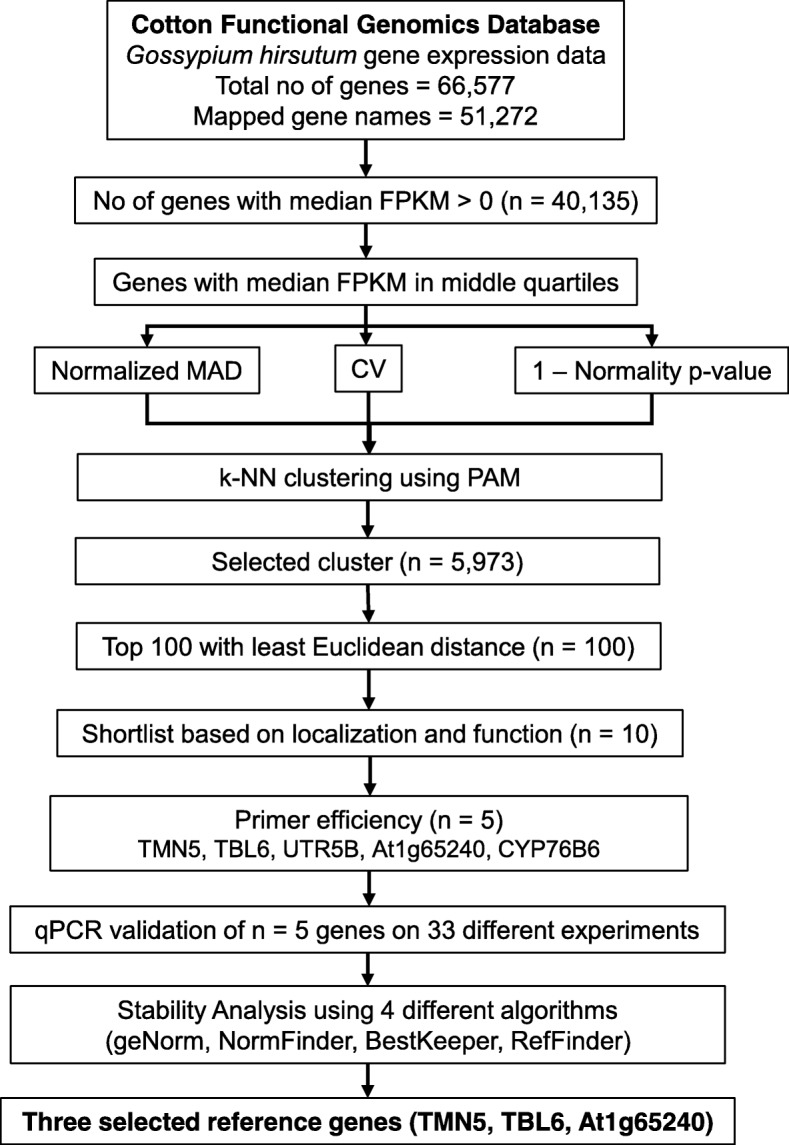
Work Flow to identify candidate reference genes with least variations and validation of the genes in experiment

Genes from Cluster 1 with least medoid value of each parameter was chosen over cluster 2 and sorted in ascending order based on the calculated Euclidean distance. The top 100 genes were further filtered out according to their GO annotation for subcellular location and molecular function. Gene products localizing to nucleus are more likely to be involved in transcription and can be subjected to variations under different experimental conditions leading to differential mRNA expression. Thus, non-cytoplasm expressed genes or genes involved in translation are not considered for further analysis. The entire workflow is summarized in Fig. 2 and the top ten genes considered for further analyses in Table 2. Two most popular reference genes used in cotton research under biotic stress, *UBQ14*, a polyubiquitin and *PP2A1,* a protein phosphatase [11], were included in the experimental validation for comparison are mentioned in Table 2.

**Table 2:** List of selected candidate reference gene for expression analysis and validation

Gene.Name	NCBI ref. seq	Description	Function
CYP76B6	XM_016861559	Geraniol 8-hydroxylase	Heme binding and oxidoreductase activity
RPK2	XM_016855096.1	LRR receptor-like serine/threonine-protein kinase RPK2	Protein Kinase activity
At1g65240	XM_016888563.1	Aspartic proteinase-like protein 2	Involved in aspartic-type endopeptidase activity
COV1	XM_016863942.1	Protein continuous vascular ring 1	Negatively regulates the differentiation of vascular tissue in the stem.
AZG1	XM_016863550.1	Adenine/guanine permease AZG1	Transports natural purines and purine analogs. Confers sensitivity to 8-azaadenine and 8-azaguanine
EMB8	XM_016834287.1	Embryogenesis-associated protein EMB8	Role in embryogenesis?
TMK3	XM_016813562.1	Receptor-like kinase TMK3	Auxin signal transduction, cell expansion, proliferation and regulation
UTR5B	XM_016900481.1	UDP-galactose/UDP-glucose transporter 5B	Sugar transporter
TMN5	XM_016895405.1	Transmembrane 9 superfamily member 5	Protein localization
TBL6	XM_016880182.1	Protein trichome birefringence-like 6	O-acetyltransferase activity
PP2A1	XM_016840233.1	Protein Phosphatase 2A	Phosphatase activity
UBQ14	XM_016867963.1	Polyubiquitin	Ubiqutination Reaction

### Selection of primers

Melting curve analysis of the top ten selected genes using pooled cDNA from various plant parts of cotton as the target, filtered five genes *TMN5, TBL6, UTR5B, AT1g65240* and *CYP76B6* that met the criteria for good primers. The use of these primers resulted in a single amplification product of expected size with the templates and no amplification (more than 35 Cq) for non-template controls (Additional file 4). Calculation of primer efficiencies using a five-fold dilution of cDNA for the five reference gene primers gave *r*^2^ > 0.97 and efficiency (E) values of 1.9–2.0 (Additional file 2 and Table 3). The rest of the primers showed very low primer efficiency.

**Table 3:** Primer sequences and efficiency of the shortlisted primers used in this study

Gene.Name	Primers (5′ to 3′)	Efficiency (%)	*R* ^2^
PP2A1	F-GATCCTTGTGGAGGAGTGGA	93.54	0.99
	R-GCGAAACAGTTCGACGAGAT		
TMN5	F-CTCACCATTCCATTACTTGTGTTG	103.24	0.97
	R-GAGGAATCTCTCTCGGGTATCT		
UBQ14	F-CAACGCTCCATCTTGTCCTT	103.74	0.99
	R-TGATCGTCTTTCCCGTAAGC		
TBL6	F-AGCAGATCCAGAGACAAGAAAG	95.14	0.99
	R-CCATTGTAGGTGCAGGTGTAT		
UTR5B	F-CGGTCTCTGCTGGTTCTTTAG	94.91	0.99
	R-TGACATGTTGTGGTTAGGATGT		
At1g65240	F-GCAAACTCTACAGCTCCCATTA	104.42	0.99
	R-GTCCAAACCCGAAGATTCCA		
CYP76B6	F-TGGCTTGGATGCCTGTTT	103.71	0.99
	R-TCGCCGTAAGTGTTGGTTAG		

### Expression analysis of candidate reference gene by qPCR

**Fig. 3:**
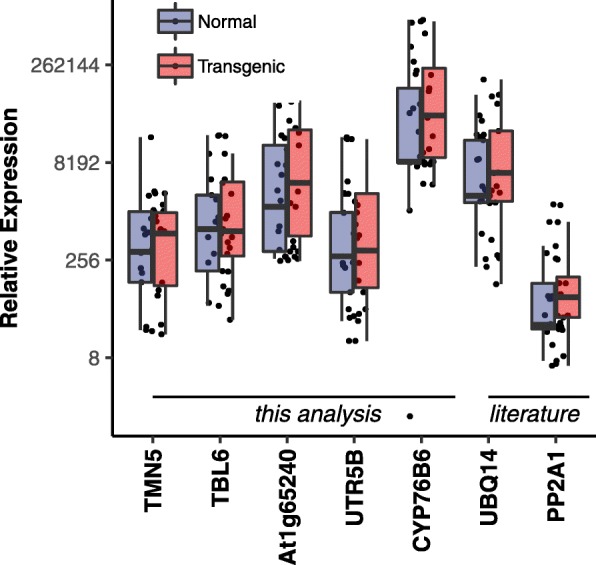
Observed expression values of candidate reference genes across normal and transgenic categories, with median expression value of each gene represented by middle horizontal lines in the box plot

Expression of the chosen candidate reference genes were analyzed across all the tissue samples (leaves, square and stem) between transgenic and non-transgenic lines (Fig. 3). Since Lepidoptera does not infect the roots, it was included only for the non-transgenic lines. RNA quality has been shown in Additional file 1. In Fig. 3, *TBL6* and *UTR5B* showed the least variation between the two categories, followed by *TMN5* and *AT1g65240*. The commonly used gene *PP2A1* although showed a lower median of expression among the study groups, yet showed greater variation between the transgenic and non-transgenic lines. Same trend can be observed with another popular gene *UBQ14*.

### Expression stability of the reference genes in different age category for various plant organs

**Fig. 4:**
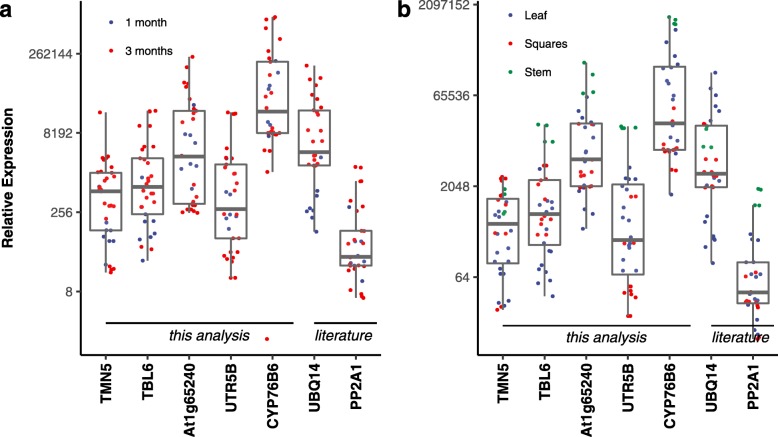
Observed expression values of candidate reference genes across (**a**) different ages of the plant and (**b**) various plant parts, with median expression value of each gene represented by middle horizontal lines in the box plot

We further analyzed the behavior of the reference genes across various plant tissues (leaf, square and stem) and different age categories (1 and 3 months old) in transgenic and non-transgenic lines with tissue samples from four biological replicates in each of the organs and age categories. It can be observed from Fig. 4a and b, that *TBL6* and *TMN5* showed a uniform expression across various tissues and age categories, suggesting the combination of these genes as optimal when analyzing the expression level of foreign or a transgene. This result is also in agreement with the geNorm analysis, which ranked both these genes in the number 1 position, with the least value of stability parameter (Additional file 5).

### Expression stability of reference genes across differential developmental stages of leaf and square tissue

**Fig. 5:**
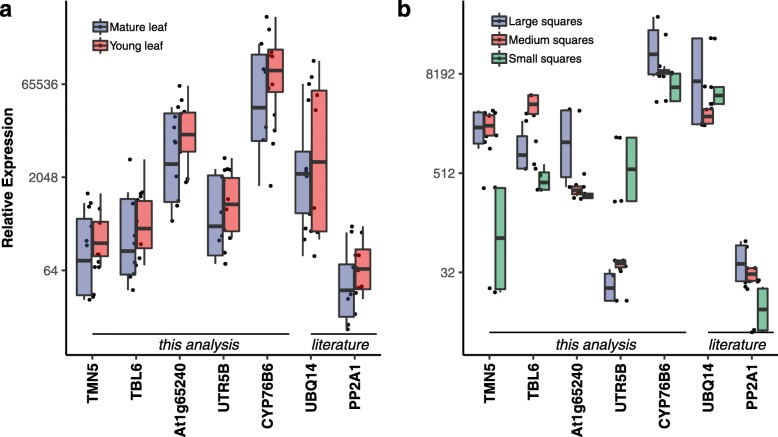
Observed expression values of candidate reference genes across (**a**) two maturity levels of the leaves and (**b**) different sizes of the squares, with median expression value of each gene represented by middle horizontal lines in the box plot

Expression analysis of the reference genes during different developmental stages of leaves showed *TMN5* (Fig. 5a) to have the least variability in median expression between the mature bottom leaves and young terminal leaves. However, the expression profile of the candidate reference gene in different developmental stages of the square tissues showed a different pattern. *CYP76B6* and *At1g65240* (Fig. 5b) showed more uniform expression in different developmental stages of the square tissue, whereas *TMN5, TBL6 and UTR5B* showed greater variability.

### Comprehensive stability analysis of candidate reference genes

**Fig. 6:**
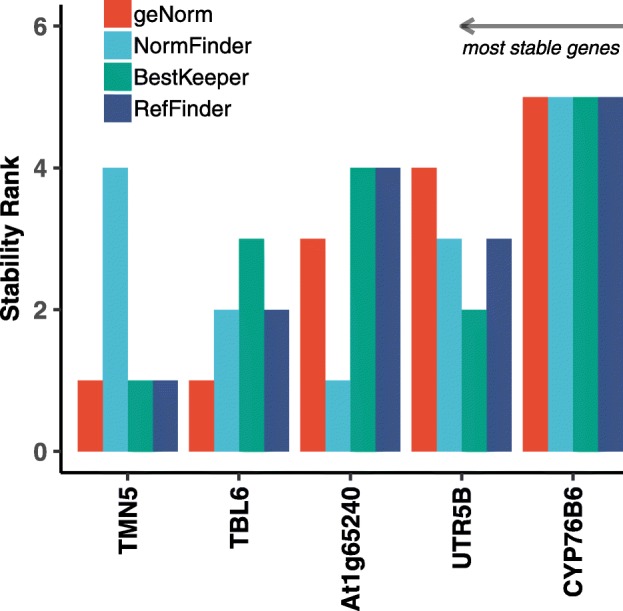
Stability Ranks of the chosen reference genes candidate using four different algorithms - geNorm, BestKeeper, NormFinder and RefFinder

Stability of candidate genes was calculated independently by BestKeeper and RefFinder from the raw C_p_ values and by geNorm and NormFinder from relative expression (Additional file 5). The geometric mean of stability ranks assigned in each algorithm was used to create a comprehensive stability ranking of all the candidate reference genes (Fig. 6). The analysis shows *TMN5* and *TBL6* to be most stable across all tissue types. However, stability analysis including commonly used reference genes from the literature indicates *PP2A1* a*t* the highest rank followed by *TMN5* and *TBL6* (Additional file 5).

## Discussion

Reliability of qPCR results are largely dependent on reference genes against which the expression level of the target gene is normalized. Hence, the choice of the reference gene is the most critical parameter while analyzing comparative gene expression studies. Most reliable reference genes should not be regulated across sample types like tissues isolated from different parts of the plant, across different age and developmental stages, various abiotic and biotic stress conditions and different genotypes. However, it is difficult to pinpoint one single reference gene to be optimal for all experimental conditions. As compiled in a review paper by Joseph et al. [11] and two recent literatures [24, 36], more than 20 reference genes have been used for expression analysis in cotton plants. Fausto et al. [47] have even used miRNA as reference genes for expression of mRNA in cotton. However, all these genes have been chosen based on their function or traditional usage in other systems. The current study is the first attempt of an unbiased genome wide search to find the most stable genes for expression analysis across various tissues and transgenic stress.

Based on the primer efficiency test, we took forward five novel genes for further experiments. Expression stability of these genes was checked by qPCR in experimental sets including different tissues, age categories, developmental stages and different genotypes. Two genes, *TMN5 and TBL6* emerged as the most overall stable genes (Fig. 6). Though there is not too much difference between *AT1g65240* and *UTR5B* in overall stability, *AT1g65240* showed much lesser variation in squares, one of the target plant organs of Cry gene expression in defence of biotic stress. Thus, in overall stability *TMN5, TBL6 and AT1g65240* emerge as clear winners*.* Among the five genes tested *CYP76B6* had the highest expression and least stability, a pattern that is observed in studies with other organisms as well [10].

A probe into the function of these genes revealed that *TMN5* encodes for Transmembrane 9 superfamily member 5 protein, which functions in protein localization to the membrane in *Arabidopsis thaliana* [48]. Interestingly, a recent genome wide search for reference genes in *Ciona savignyi*, a kind of mollusc, also has identified *TMN* to be a stable gene [10]. *TBL6* encodes for trichome birefringence-like 6 which is involved in acetyltransferase activity in *Arabidopsis* [49]. The protein coded by *AT1g65240* gene, Aspartic proteinase-like protein has been shown to be expressed in seed pods and proposed to play a role in the processing and degradation of the storage proteins in the seeds [50] and thus explaining its high expression levels in the square tissues. Similarly, *CYP76B6*, geraniol hydroxylase, belongs to the family of CYP76 coding for cytochrome P450 enzyme, which catalyzes the single or double oxidation of all linear mono-terpenols, derivatives of which are particularly found in flower, fruit and young leaves [51]. The protein encoded by *UTR5B* is involved in carbohydrate transport [52].

Two genes from the literature *PP2A1* and *UBQ14,* when compared on the same experimental platforms with the five genes found in this study, revealed that the lower expressing *PP2A1* was comparable to *TMN5* and *TBL6.* The protein encoded by *PP2A1* is the catalytic subunit of protein phosphatase 2A [26], a trimeric holoenzyme, ubiquitously expressed serine/Threonine protein phosphatase, conserved throughout eukaryotes. Though the holoenzyme, among many functions, is involved in regulation of pathogen response [53], the catalytic subunit showed stability between the non-transgenic and transgenic cotton plants.

As documented in this study, most stable genes found by this analysis have very diverse functions. Thus, our traditional idea of selecting reference genes based on function does not hold good any longer.

This is the first time, validation of reference genes to be used in qPCR was done combining analysis of RNA-seq data and experimental validation for non-transgenic and transgenic cotton plants. This study becomes important as the transgenic cotton plants account for 95% of the cotton grown in the total cotton growing areas in India [54] and periodic estimation of transgene expression levels is a critical checkpoint, to ascertain the functionality of these transgenic crops. However, this study only covers the expression stability of the reference genes under normal field conditions and does not include the effect of biotic and abiotic stress conditions on the candidate reference genes.

## Conclusion

The present study has employed a data-driven approach for the identification and experimental validation of reference genes to be used for qPCR studies in transgenic and non transgenic lines of *Gossypium hirsutum*. Out of the five new candidate reference genes *TMN5*, *TBL6*, *At1g65240*, *CYP76B6* and *UTR5B* analyzed, the first three show stable levels of expression in all the conditions tested in our experiments and hence stands out to be potential reference genes in cotton species *G. hirsutum.*

## Methods

Workflow for this study (Fig. 2) comprised of two broad sections – (i) The statistical analysis of publicly available RNA-seq data to identify candidate reference genes with the least variations in expression and (ii) experimental validation of the selected reference genes.

### Gene expression data acquisition

RNA-seq Fragments per kilobase per million reads (FPKM) values for *G. hirsutum* were downloaded from the Cotton Functional Genomics Database (CottonFGD) [40], which to our knowledge was the only publicly available dataset with RNA-seq expression data for *G. hirsutum* genes. FPKM values inherently contained two normalization factors - (i) for differences in transcript lengths across genes and (ii) for differences in library sizes across experiments. Since in this analysis our aim was to identify stable genes showing least variations across samples, further normalization was not carried out to remove such variations. Recent published literatures on similar transcriptomic analysis for other organisms [10, 21–23] have also used FPKM or TPM values without further normalization.

CottonFGD contained gene expression data of 66,577 genes across 55 different experimental conditions in different experimental groups such as seed germination (time-series and organ comparison), tissue and organ, ovule development (time-series) and stress experiment (time series for hot, cold, salt and PEG treated samples along with control groups). We downloaded the Joint Genome Institute (JGI) assembly [41] annotation of *G. hirsutum* from the same database and shortlisted the genes only with mapped gene names and chromosome locations.

In any gene expression experiment, low-expressing genes often create problems for downstream analysis as expression values of such genes are often indistinguishable from sampling noise. To eliminate such genes from the analysis, we used a filtering criterion of median FPKM value > 0 (or in other words non-zero FPKM value in at least 28 out of 55 samples). Genes those passed the above criteria was selected for the statistical analysis (Fig. 2).

### Statistical analysis

To assess the stability of a gene, two measures were adopted – (i) CV $=\overline{x}/{\sigma}_x$ where $\overline{x}$ and *σ*_*x*_ are mean and standard deviation of a variable *x* respectively and (ii) the normality *p*-value measured by the Shapiro-Wilks Test (*p*-value < 0.05 indicates the distribution to be away from Normal) [18]. CV, albeit most frequently used, is affected by outliers and hence fails to be a robust measure. To address this, a third parameter – Median Absolute Deviation ($\mathrm{MAD}=\kern0.5em median\kern0.75em \left|x-\hat{x}\right|$ where $\hat{x}$ is the median of *x*) [42] was used after normalization with the median. MAD is a measure of the spread of the distribution and is based on medians, thus less susceptible to deviations by outliers.

An ideal set of reference genes should have low or similar statistical variation across samples (represented by low values of CV and MAD) and should behave as much like a normal distribution (high value of normality p-value or low values of 1 – p-value). Therefore, a k-medoids clustering of genes was clustered based on the values of the three statistical parameters – CV, MAD (normalized to respective z-scores) and 1 – p-value using the PAM (Partitioning around Medoids) algorithm originally proposed by Kaufman and Rousseeuw [43]. Medoid based clustering approach was chosen over the more commonly used k-means method to reduce the effect of outlier genes in cluster determination. The optimal number of clusters required is calculated using the Silhouette graphical method of Rosseeuw [44]. Cluster having the lowest medoid values for each of the three parameters was selected and genes in the cluster were ranked using the Euclidean distance $d=\sqrt{CV^2+{MAD}^2+{\left(1-p\right)}^2}$ (all parameters replaced by their z-scores) in this three-parameter hyperspace. Top hundred genes with the least values of Euclidean distance were selected and their subcellular location and function were analyzed using Gene Ontology annotation.

### Selection of commonly used reference genes from literature

The most commonly used reference genes were shortlisted from literature keeping in mind their frequencies of usage in the published scientific literature on cotton from 2016 till recent. There were no unique keywords that were used by researchers to report reference genes in expression studies. Hence, a very broad methodology was adopted in which all articles in PubMed were searched for the occurrence of any of the terms “reference gene” or “control gene” or “housekeeping gene” along with co-occurrence of the term *Gossypium hirsutum* anywhere in the title and abstract. The obtained abstracts were manually curated to find the relevant articles that described studies on reference genes specifically in the context of the cotton plant.

### Plant material

Experiments were performed using 1 and 3 months old transgenic (Bollgard II, Monsanto 15,985, an insect resistant line) and non-transgenic lines of *Gossypium hirsutum* plants grown under normal field conditions, with a temperature range of 25 °C ± 4 °C and natural photoperiod. During the course of the study, there was no biotic or abiotic stress applied to the plants. The organs used were stem, terminal young leaves, bottom mature leaves and squares in different developmental stages (Small: pin and match head squares, medium: square growth midpoint and large: candle squares). Roots were used only from non-transgenic plant. Replicates were decided as per Artico et al. [26]. Briefly, the material was harvested from three different cotton plants to obtain one pool. The procedure was repeated with three distinct plants in order to obtain a second pool, the biological replicate. A total of 33 experimental categories from transgenic and non-transgenic lines were prepared comprising of leaf, squares, root and stem tissues. For each of the statistical analysis performed in various experimental categories, four replicates of each kind of tissues were analyzed. Total RNA was freshly extracted from all the tissues and stored at -80 °C.

### Total RNA isolation and cDNA synthesis

Total RNA was extracted from fresh tissues as described previously [45] with slight modifications. Briefly, fresh tissues were homogenized to a fine paste in pre-cooled mortar and pestle using 10 ml of extraction buffer (400 μl of β–mercaptoethanol and 4% polyvinyl pyrrolidone) per gram of the plant tissue. An equal volume of water saturated-phenol, chloroform and isoamyl alcohol, at a ratio 25:24:1, was added, mixed thoroughly and centrifuged. The separated aqueous phase was extracted with chloroform-isoamyl alcohol, followed by the addition of lithium chloride (LiCl) to a final concentration of 3 M. After overnight incubation at − 20 °C, the RNA precipitate was re-suspended in 2 M LiCl, centrifuged and washed with 70% ice-cold ethanol. Pellet was air-dried and dissolved in 500 μl of sterile water. The RNA quality and purity were determined using a NanoDrop™ 2000/2000c Spectrophotometer (Thermo Scientific) and the integrity of RNA was checked by 1% agarose gel electrophoresis and ethidium bromide staining (Additional file 1).

cDNAs were synthesized by adding 50 μM of Oligo (dT) primer, 10 mM of dNTPs (MBT079, HiMedia), AMV RT (NEB, #M0277S), 1X First Strand Buffer, RNase inhibitor (Applied Biosystems, N8080119) to 1 μg of total RNA. The mixture was incubated at 42 °C for 1 h following the manufacturer’s instruction. Inactivation of the reverse transcriptase was done by incubating the mixture at 80 °C for 5 min and the cDNA solution was stored at − 20 °C.

### Real-time quantitative polymerase chain reaction (qPCR)

#### Primer design

Primers were designed from the top ten selected genes (Table 2) using the primer Quest tool (https://eu.idtdna.com/pages/tools/oligoanalyzer, 24 May 2019, Integrated DNA Technologies, Inc.) for real-time PCR from Integrated DNA technologies using the criteria that the amplified products range from 90 to 110 bp with a Tm of 62 ± 1 °C. Melt curve analysis of the top 10 selected candidate genes was done and primers showing a single amplicon was chosen for further experiments. The primer sets efficiencies of each primer pair were determined by the standard curve method using serial dilutions of the pooled cDNA, using the formula, Efficiency (%) = (10 ^-1/slope^ − 1) × 100 (Table 3 and Additional file 2).

qPCR was carried out on Roche LightCycler 480 II instrument using KAPA SyBr green Universal kit (Sigma, #KK4600) using a 96 well or 384 well optical plates (Roche LightCycler® 480 Multiwell Plate 96 or 384, clear, C0687653 384 or C1468659). Reaction mixtures contained 1 μl of diluted cDNA in water (1:1), 0.2 μM of each primer, 2.5 μl of KAPA SyBr Green mix, 1.3 μl of water in a total volume of 5 μl. Reaction mixtures were incubated for 10 s at 95 °C followed by 45 amplification cycles of 10 s at 95 °C; 15 s at 60 °C and 15 s at 72 °C. For each of the sample, the qPCR was performed in technical replicates of three. Three negative controls in which the cDNA was replaced with nuclease free water were also included for each of the primer pair.

#### Analysis of gene expression stability

From Quantification cycle (Cq) values of each gene in qPCR experiments, mean of non-template control (NTC) Cq values were subtracted to obtain ΔC_q_ = C_q_ (sample) – Mean C_q_ (NTC) and relative expression as E^-ΔCq^ for each replicate, where E is the primer efficiency of each gene obtained from standard curve. Geometric mean of expression values of replicates are plotted for the chosen reference genes across different samples as depicted in results.

To analyze stability of expression, we used three distinct algorithms geNorm [13], NormFinder [14] and BestKeeper [15] independently and also the web-based RefFinder [19] tool that integrates all these three algorithms plus the DeltaCT method. geNorm algorithm was run using the SLqPCR R package [46], whereas author-supplied R package or Excel worksheet was used for NormFinder and BestKeeper respectively. Mean Cq values for each gene for all 33 experiments were used as input for BestKeeper and RefFinder, whereas for geNorm and NormFinder the relative expression values were used. Since NormFinder uses a model-based approach to quantify inter- and intra-group variations, the normal and transgenic samples are used as two groups for NormFinder analysis.

Results of BestKeeper algorithm, used independently or as part of RefFinder were comparable. However, the rankings differed since BestKeeper uses correlation coefficient with BestKeeper index as ranking parameter, whereas its RefFinder implementation uses standard deviation to rank the genes. Results of geNorm and Normfinder analysis, independently or within ReFinder tool were not compared as they used different inputs. Comprehensive stability rank of each gene was calculated as the geometric mean of stability rank given by each method.

## Additional files

Additional file 1: Gel image of all the RNA samples used in the study. (DOCX 17394 kb)

Additional file 2: Primer pair efficiency. (PDF 211 kb)

Additional file 3: List of Genes selected in the study. The list of genes in cluster 2 (sheet 1) and cluster 1(sheet 2) top 100 Genes in the selected cluster 1, sorted in ascending order based on the calculated Euclidean distance (sheet 3) and the selected 10 (sheet 4) genes are listed. (XLSX 1375 kb)

Additional file 4: Melt curves of the final selected primer. (PDF 861 kb)

Additional file 5: Stability analysis of the candidate reference genes using different algorithms. (XLSX 69 kb)

## Data Availability

The datasets analyzed during the current study are available from the corresponding author on reasonable request. All data generated or analyzed during this study are included in this published article [and its Additional files].

## Abbreviations

At1g65240: Aspartic proteinase-like protein 2
AZG1: Adenine/guanine permease AZG1
cDNA: Complementary DNA
COV1: Protein continuous vascular ring 1
Cq: Quantification cycle
CV: Coefficient of variation
CYP76B6: Geraniol 8-hydroxylase
DNA: Deoxyribo nucleic acid
EMB8: Embryogenesis-associated protein EMB8
FPKM: Fragments per kilobase of transcript per million mapped reads
GO: Gene Ontology
JGI: Joint Genome Institute
MAD: Median Absolute Deviation
NCBI: National Center for Biotechnology Information
NTC: Non-template control
PAM: Partitioning around Medoids
PEG: Polyethylene glycol
PP2A1: Protein phosphatase 2A
qPCR: Quantitative real-time polymerase chain
RNA: Ribo nucleic acid
RPK2: LRR receptor-like serine/threonine-protein kinase RPK2
RT-PCR: Reverse transcription polymerase chain reaction
TBL6: Protein trichome birefringence-like 6
TMK3: Receptor-like kinase TMK3
TMN5: Transmembrane 9 superfamily member 5
UBQ14: Polyubiquitin
UTR5B: UDP-galactose/UDP-glucose transporter 5B
