# Supplementary material for: Genome wide search to identify reference genes candidates for gene expression analysis in Gossypium hirsutum
Source: BMC Plant Biol. 2019 Sep 14;19:405. doi: 10.1186/s12870-019-1988-3 (PMC6744693; doi:10.1186/s12870-019-1988-3)
Supplement: Supplementary file 1 — Gel image of all the RNA samples used in the study. (DOCX 17394 kb) [file 12870_2019_1988_MOESM1_ESM.docx]

**RNA Quality Check (1% agarose gel)**


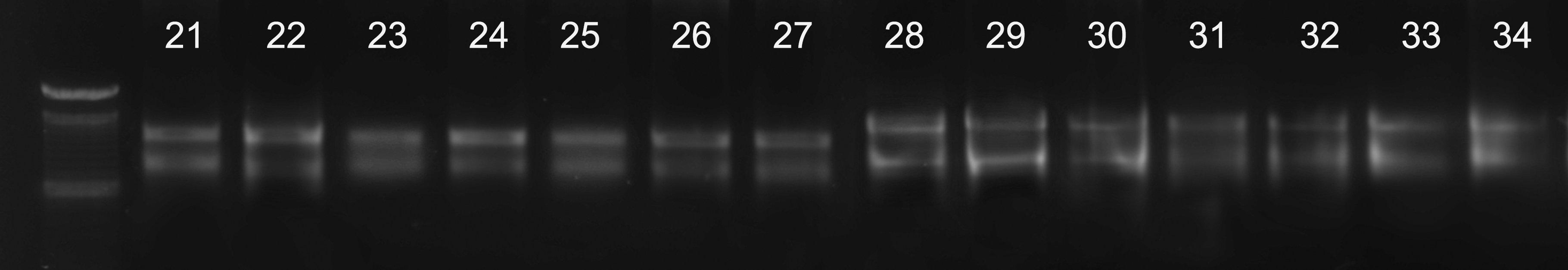

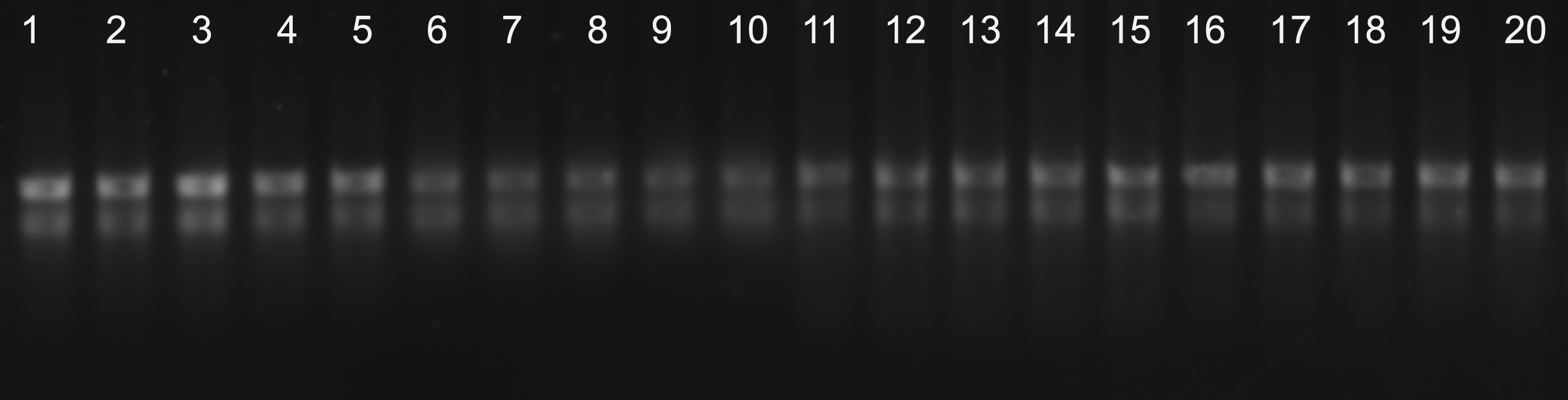


1. One Month, Young Leaves, Transgenic: (Pooled Plant T1, T2, T3)
2. One Month, Young Leaves, Transgenic: (Pooled Plant T4, T5, T6)
3. One Month, Young Leaves, Non-Transgenic: (Pooled Plant NT1, NT2, NT3)
4. One Month, Young Leaves, Non-Transgenic: (Pooled Plant NT4, NT5, NT6)
5. One Month, Mature Leaves, Transgenic: (Pooled Plant T1, T2, T3)
6. One Month, Mature Leaves, Transgenic: (Pooled Plant T4, T5, T6)
7. One Month, Mature Leaves, Non-Transgenic: (Pooled Plant NT1, NT2, NT3)
8. One Month, Mature Leaves, Non-Transgenic: (Pooled Plant NT4, NT5, NT6)
9. Three Month, Young Leaves, Transgenic: (Pooled Plant 3/T1, 3/T2, 3/T3)
10. Three Month, Young Leaves, Transgenic: (Pooled Plant 3/T4, 3/T5, 3/T6)
11. Three Month, Young Leaves, Non-Transgenic: (Pooled Plant 3/NT1, 3/NT2, 3/NT3)
12. Three Month, Young Leaves, Non-Transgenic: (Pooled Plant 3/NT4, 3/NT5, 3/NT6)
13. Three Month, Mature Leaves, Transgenic: (Pooled Plant 3/T1, 3/T2, 3/T3)
14. Three Month, Mature Leaves, Transgenic: (Pooled Plant 3/T4, 3/T5, 3/T6)
15. Three Month, Mature Leaves, Non-Transgenic: (Pooled Plant 3/NT1, 3/NT2, 3/NT3)
16. Three Month, Mature Leaves, Non-Transgenic: (Pooled Plant 3/NT4, 3/NT5, 3/NT6)
17. Three Month, Stem, Transgenic: (Pooled Plant 3/T1, 3/T2, 3/T3)
18. Three Month, Stem, Transgenic: (Pooled Plant 3/T4, 3/T5, 3/T6)
19. Three Month, Stem, Non-Transgenic: (Pooled Plant 3/NT1, 3/NT2, 3/NT3)
20. Three Month, Stem, Non-Transgenic: (Pooled Plant 3/NT4, 3/NT5, 3/NT6)
21. Three Month, Small Square, Transgenic: (Pooled Plant 3/T1, 3/T2, 3/T3)
22. Three Month, Small Square, Transgenic: (Pooled Plant 3/T4, 3/T5, 3/T6)
23. Three Month, Small Square, Non-Transgenic: (Pooled Plant 3/NT1, 3/NT2, 3/NT3)
24. Three Month, Small Square, Non-Transgenic: (Pooled Plant 3/NT4, 3/NT5, 3/NT6)
25. Three Month, Medium Square, Transgenic: (Pooled Plant 3/T1, 3/T2, 3/T3)
26. Three Month, Medium Square, Transgenic: (Pooled Plant 3/T4, 3/T5, 3/T6)
27. Three Month, Medium Square, Non-Transgenic: (Pooled Plant 3/NT1, 3/NT2, 3/NT3)
28. Three Month, Medium Square, Non-Transgenic :(Pooled Plant 3/NT4, 3/NT5, 3/NT6)
29. Three Month, Large Square, Transgenic: (Pooled Plant 3/T1, 3/T2, 3/T3)
30. Three Month, Large Square, Transgenic: (Pooled Plant 3/T4, 3/T5, 3/T6)
31. Three Month, Large Square, Non-Transgenic: (Pooled Plant 3/NT1, 3/NT2, 3/NT3)
32. Three Month, Large Square, Non-Transgenic: (Pooled Plant 3/NT4, 3/NT5, 3/NT6)
33. Root Tissue, Non-Transgenic: (Pooled Plant 3/NT1, 3/NT2, 3/NT3)
34. Root Tissue, Non-Transgenic: (Pooled Plant 3/NT4, 3/NT5, 3/NT6)
