# Supplementary figures and images for: Genome wide search to identify reference genes candidates for gene expression analysis in Gossypium hirsutum
Source: BMC Plant Biol. 2019 Sep 14;19:405. doi: 10.1186/s12870-019-1988-3 (PMC6744693; doi:10.1186/s12870-019-1988-3)

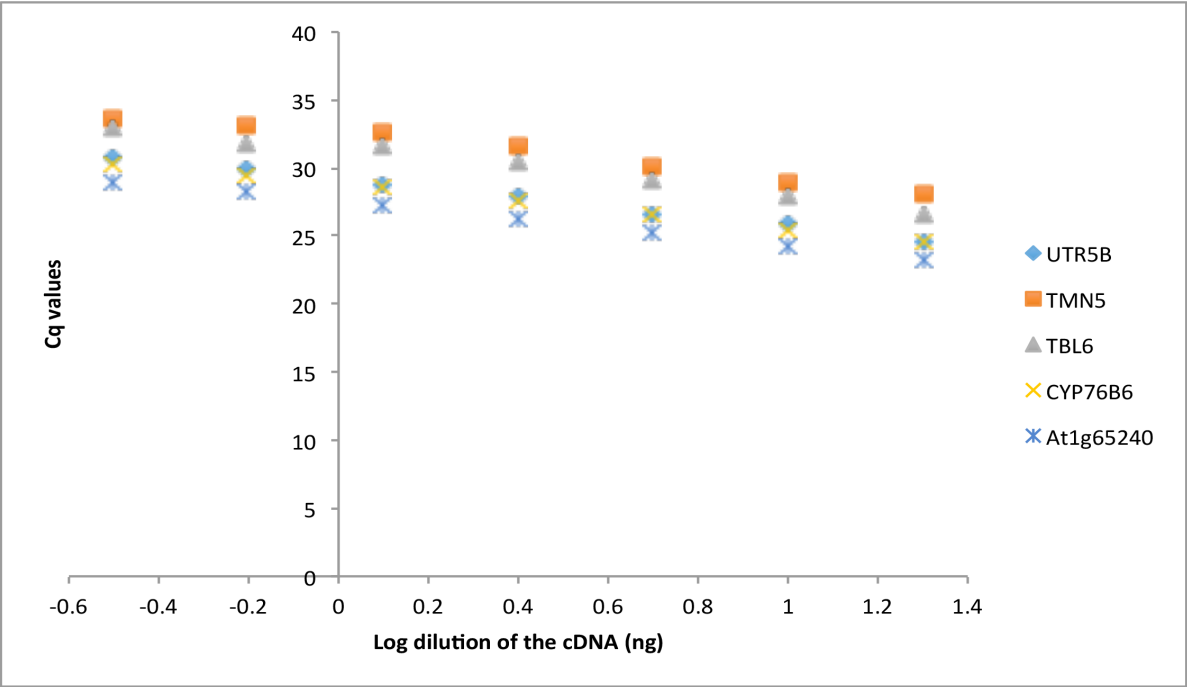

Amplification plot of all the primer pairs

Supplement: Supplementary file 2 — Primer pair efficiency. (PDF 211 kb) [file 12870_2019_1988_MOESM2_ESM.pdf]

## Melt curves of Primers used in the study

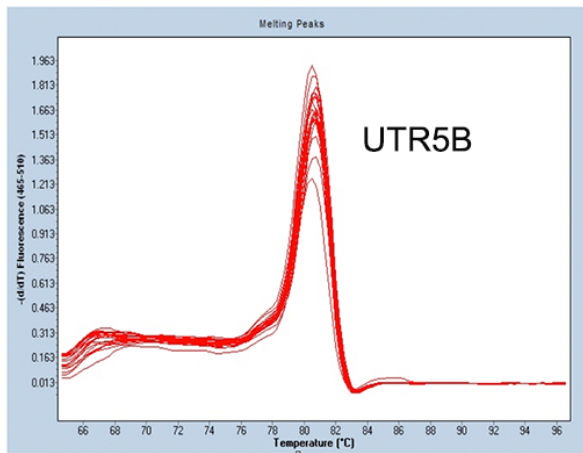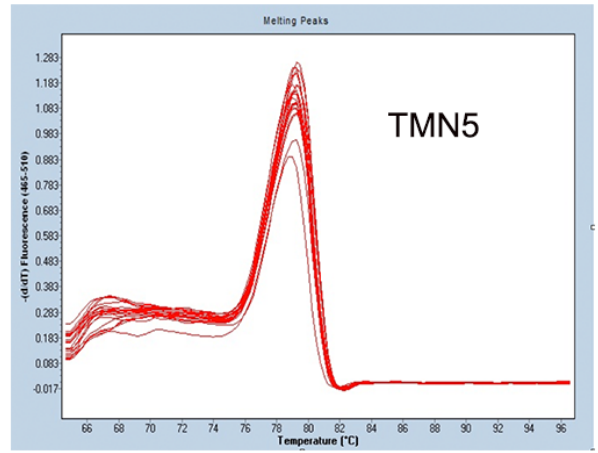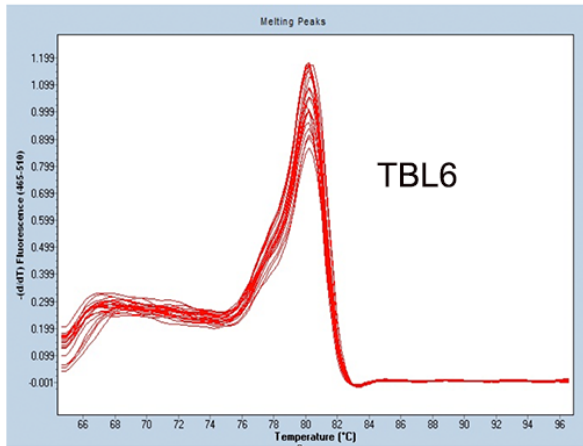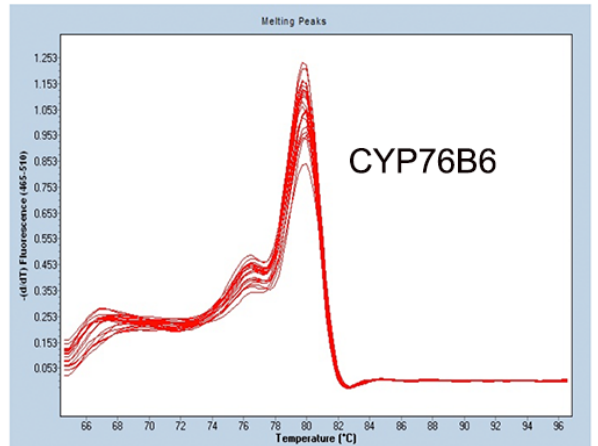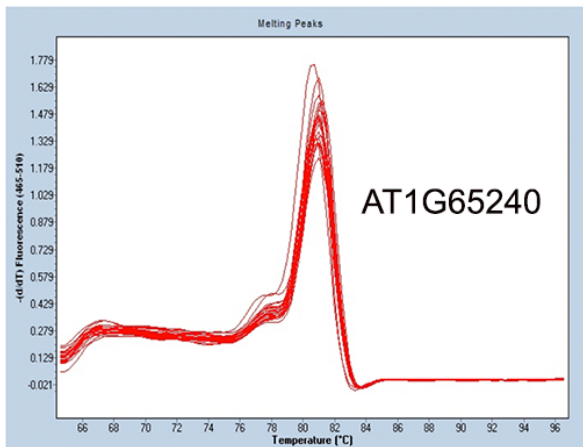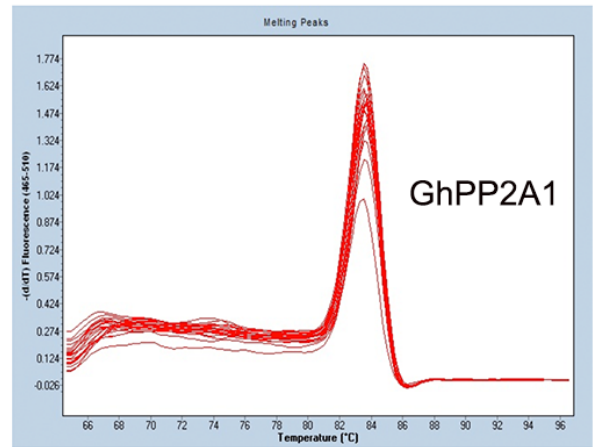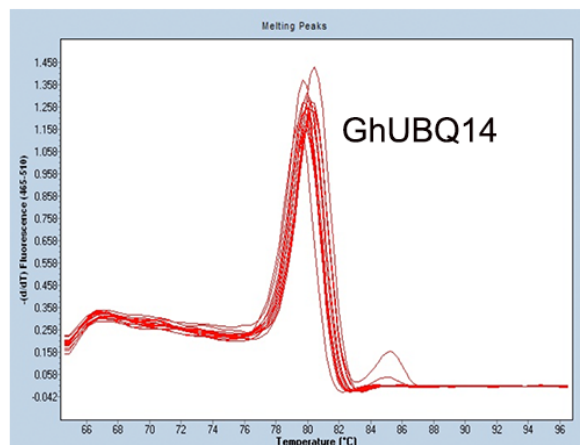

Supplement: Supplementary file 4 — Melt curves of the final selected primer. (PDF 861 kb) [file 12870_2019_1988_MOESM4_ESM.pdf]
